# Editorial: The Association of Other Autoimmune Diseases in Patients With Thyroid Autoimmunity

**DOI:** 10.3389/fendo.2018.00540

**Published:** 2018-09-13

**Authors:** Alessandro Antonelli, Salvatore Benvenga

**Affiliations:** ^1^Department of Clinical and Experimental Medicine, University of Pisa, Pisa, Italy; ^2^Department of Clinical and Experimental Medicine, Section of Endocrinology, University of Messina, Messina, Italy; ^3^Master Program on Childhood, Adolescent and Women's Endocrine Health, University of Messina, Messina, Italy; ^4^Interdepartmental Program on Molecular & Clinical Endocrinology, and Women's Endocrine Health, University Hospital, A.O.U. Policlinico Gaetano Martino, Messina, Italy

**Keywords:** autoimmune thyroid diseases, rheumatological autoimmune disorders, Graves' disease, Hashimoto's thyroiditis, thyroid autoantibodies, postpartum, epigenetics, environmental factors

Autoimmune thyroid diseases (AITD) derive from a dysregulation of the immune system causing an immune attack on the thyroid, and involve two main clinical presentations: Graves' disease (GD) and Hashimoto's thyroiditis (AT) (1).

Recently, a significant increase of the prevalence of other autoimmune disorders in AT patients has been shown (by a large prospective, case-control, study) for the following diseases: vitiligo (Vit), chronic autoimmune gastritis (CAG), sjogren disease (SS), rheumatoid arthritis (RA), multiple sclerosis, polymyalgia rheumatica (Polym), celiac disease, diabetes, systemic lupus erythematosus (SLE), sarcoidosis (S), alopecia, psoriathic arthritis (PsA), systemic sclerosis (SSc), and hepatitis C virus (HCV)-related mixed cryoglobulinemia (MC); and a near significant prevalence has also been shown for Addison's disease and ulcerative colitis (2). Furthermore, the association of three autoimmune disorders was observed in AT patients (the most frequent associations were AT + CAG + Vit and AT + CAG + Polym) (2).

Moreover, in a large number (*n* = 2,791) of UK patients with GD a significant association with RA, pernicious anemia, SLE, Addison's disease, celiac disease, and Vit has been shown. A relative “clustering” of AITD in the index case with parental AITD was present. Furthermore relative risks for most other coexisting autoimmune disorders were strongly increased among parents of index cases (3).

Conversely, patients affected with the above mentioned autoimmune disorders are more frequently affected by AITD (1).

In this Research Topic, constituted by eighteen papers, we review and discuss new evidence about the association of other autoimmune diseases in patients with AITD, providing a stimulating overview of the present knowledge.

The paper by Coppedè discusses the increasing evidence about the importance of epigenetic modifications (such as changes in DNA methylation, non-coding RNA molecules-mediated gene silencing, and covalent modifications of histone tails) in the pathogenesis of AITD, as a possible result of environmental injuries that trigger these disorders.

The autoimmune/inflammatory syndrome induced by adjuvants (ASIA), presented by Shoenfeld and Agmon-Levin (4), is constituted by different autoimmune conditions induced by the exposure to various adjuvants, that are present in many vaccines, and induce immune reactions (5). The review by Watad et al. summarizes the current knowledge on ASIA syndrome presented as endocrinopathies, concentrating on adjuvants-associated AITD.

Fallahi et al. provide evidence on the association of SSc and thyroid disorders. The majority of the reviewed papers report an association among SSc, hypothyroidism, and AT (and few cases of GD). Moreover, a high prevalence of thyroid cancer, in SSc patients with AT, has been observed.

Increased incidence of AT and hypothyroidism has been shown in SLE patients, particularly in females. The review by Ferrari et al. suggests female patients with SLE, showing a high risk profile of AITD [a small and hypoechoic thyroid, a thyroid-stimulating hormone (TSH) in the upper quartile of the normal range, and antithyroid peroxidase antibodies (AbTPO) positivity], should be periodically re-evaluated for thyroid dysfunction, and when necessary due treatments.

Mixed cryoglobulinemia is the most important systemic HCV-related extrahepatic disease (6). HCV is a hepato- and lymphotropic virus responsible for many autoimmune/lymphoproliferative and/or neoplastic disorders and a high incidence of new cases of AT and thyroid dysfunctions have been reported in MC patients (6). The paper by Ferri et al. describes the prevalence and the clinical and serological features associated with thyroid involvement, in particular AT and papillary thyroid cancer, in patients affected by chronic HCV infection in presence/absence of cryoglobulinemic vasculitis.

Regarding RA, many studies have reported that the therapy with biological antirheumatic agents (BAAs) are able to modify the inflammatory response both in RA such as in AITD. The paper by Bliddal et al. investigates how the use of such agents affect the thyroid function and autoimmunity in RA patients.

Psoriasis (PsO) is a chronic autoimmune skin disease, and PsA (a chronic inflammatory arthritis) is present in about 30% of patients with PsO. An increased rate of new cases of patients with positive AbTPO, hypothyroidism, thyroid dysfunctions and appearance of a small thyroid with signs of hypoechogenicity, overall in females, has been reported in PsA (7). PsO, PsA, and their association with AITD have been evaluated by Ruffilli et al. suggesting that a thyroid screening should be done routinely in PsA female patients with an elevated risk pattern (a TSH level in the upper quartile of the reference range, AbTPO positivity, a small and hypoechogenic thyroid).

Also the association of S and thyroid autoimmunity has been shown by different studies in a wide range of variability, particularly in the female gender (8). Fazzi et al. provide evidence about the possible association between S and thyroid autoimmunity.

Sjögren's syndrome and AITD frequently coexist in clinical practice, leading to a complex overlapping disorder that represents a particular example of the expression of heterogeneity in patients with autoimmune disorders. Baldini et al. provide a critical overview of the recent literature on the pathogenesis and clinical features of SS-AITD overlapping disease.

Non-segmental Vit is an autoimmune disorder arising from an autoimmune response against melanocytes in the skin, and it is often associated with other autoimmune disorders (9). Baldini et al. describe the clinical association between Vit and AITD and evaluate the possible common molecular pathways involved in their pathogenesis.

Lichen planus (LP) and lichen sclerosus (LS) are cutaneous-mucous disorders, too. Their etiology is unknown, clinical and histological data suggest an autoimmune pathogenesis; however only few papers evaluated their association with AITD. Guarneri et al. review the scientific literature about the correlation between AITD and lichen, and the genetic risk factors in common between these conditions.

Myasthenia gravis (MG) is a neuromuscular disease caused by a deficient transmission of the nerve impulse to muscles, leading to muscle weakness and exaggerated fatigue, that is mediated by autoantibodies. AITD and MG are frequently associated; their etiology is multifactorial and depends on genetic and environmental factors. The review by Lopomo and Berrih-Aknin focuses on AITD and MG, their common features and their diversities.

The association between CAG and AITD has been first shown at the beginning of 1960s, and recently has been included in polyglandular autoimmune syndrome type IIIb (10). Cellini et al. summarize the most recent achievements on this peculiar association.

Santoro et al. evaluate the association between AT and glomerulopathies. IgA nephropathy, membranous nephropathy, membranoproliferative glomerulonephritis, focal segmental glomerulosclerosis, amyloidosis, minimal change disease, and antineutrophil cytoplasmic autoantibody (ANCA) vasculitis are renal disorders frequently observed in AT. Different hypotheses have been reported about the association between AITD and glomerulopathies. A probable mechanism at the basis of the association between ANCA vasculitis and AT is the cross-reactivity between antigens in presence of a genetic predisposition.

Moreover, this Research Topic evaluates also thyroid autoimmunity during the complex period after childbirth, that is critical for the *de novo* appearance or exacerbation of autoimmune diseases, and AITD. Di Bari et al. describe the postpartum thyroid diseases that consist of postpartum thyroiditis (PPT), but also GD and non-autoimmune thyroiditis.

Postpartum mood disorders are a common form of maternal psychiatric morbidity, owing to the rapid endocrine and psychological alterations in the postpartum period (11). Le Donne et al. discuss the interaction between thyroid autoantibodies and mood disorders.

Antiphospholipid syndrome (APS) is an autoimmune disorder manifesting as recurring venous or arterial thrombosis that can induce complications during pregnancy and is associated with the presence of persistent antiphospholipid (aPL) autoantibodies. Versini conducts a literature review on the present data on aPL/APS and AITD, and particularly on the role of this association in obstetrical complications.

The exact pathogenetic mechanisms underlying the above reported disorders are not completely known. The effect of genetic on the association of different autoimmune disorders has been shown, as: (a) there is significant clustering of AITD within families (40–50% of AT patients have another family member with AT) (12); (b) a clear evidence comes from twin studies for GD (13) and AT (14) with concordance rates of 30–40% in monozygotic twins and 0–7% in dizygotic twins.

Moreover, new recent insights in genome-wide association studies (GWAS) about autoimmune and immune-mediated diseases have increased the knowledge of the pathogenesis underlying these disorders (15), suggesting a common genetic susceptibility (15).

Environmental factors [selenium, and vitamin D deficiency, high iodine intake, exposure to radiation (owing to nuclear fallout or medical irradiation)] are of particular importance for the appearance of AITD in susceptible subjects (16). Cigarette smoking is associated with GD and Graves' ophthalmopathy (GO), although it reduces the risk of hypothyroidism and thyroid autoimmunity. Also viral infections can drive AITD, in particular human parvovirus B19 (EVB19) and HCV. Regarding the various existing chemical contaminants, pesticides and halogenated organochlorines differently disrupt thyroid function. Polychlorinated biphenyls and their metabolites and polybrominated diethyl ethers bind to thyroid transport proteins (i.e., transthyretin), displace thyroxine, in this way disrupting thyroid function. Considering drugs, interferon- and medicines containing iodine have been associated with AITD. These environmental issues have been presented in a recent paper by Ferrari et al. (16).

A prevalent Th1 immune pattern has been shown in patients with AT, GD, GO (17, 18), type 1 diabetes, SLE, SSc, RA, MC, and others (19), in the initial phase of these disorders. Furthermore, in GD, GO, SLE, MC (and others) a Th1 prevalence has been shown in the active phase, that switches to a Th2 profile in the inactive phase (20, 21) of the disease. So it has been hypothesized that the influence of genetic and environmental factors could lead to the onset of autoimmune phenomena in different organs in the same subject (1), characterized by predominance of a Th1 immune pattern at the beginning, and in the active phase of these disorders.

In conclusion, an association of other autoimmune diseases in patients with thyroid autoimmunity has been shown, and this Research Topic provides an extensive update of the literature, and suggests interesting points for new investigations.

## Author contributions

AA and SB gave substantial contribution in writing the article, and revised it critically for important intellectual content. AA and SB agreed to be accountable for all aspects of the work in ensuring that questions related to the accuracy or integrity of any part of the work are appropriately investigated and resolved.

### Conflict of interest statement

The authors declare that the research was conducted in the absence of any commercial or financial relationships that could be construed as a potential conflict of interest.
